# The effect of rollover footwear on the rollover function of walking

**DOI:** 10.1186/1757-1146-6-24

**Published:** 2013-07-09

**Authors:** Saeed Forghany, Christopher J Nester, Barry Richards

**Affiliations:** 1 School of Health Sciences, University of Salford, Salford M6 6PU, England; 2 Musculoskeletal Research Centre, Isfahan University of Medical Sciences, Isfahan, Iran

**Keywords:** Rollover footwear, Rollover function, Rollover shape, Shoe radii

## Abstract

**Background:**

Rollover footwear is assumed to provide an enhanced surface over which the body can roll more easily. The aim of this study was to investigate the effects of rollover footwear on the rollover function of walking.

**Methods:**

Twenty subjects walked in three conditions: (i) a MBT shoe (Masai Barefoot Technology) characterized by a stiff sole rounded in the anterior–posterior direction; (ii) alternative rollover shoe (a prototype of Scholl STARLIT) characterized by a stiff sole rounded in the anterior–posterior direction; (iii) a flat control shoe. Data on the lower limb kinematics and ground reaction force were collected. The rollover function of walking was characterized using the radii of lower limb rollover shapes and duration of terminal double limb support. These data were compared between the three shoe conditions and the relationship between the radii of the curved shoe sole and the radii of the rollover shapes investigated.

**Results:**

The radii of the whole and middle part of foot–shoe, ankle-foot and knee–ankle–foot rollover shapes were significantly smaller (i.e. more curved) for MBT (ranging from 12% to 81% smaller) and the rollover shoe (ranging from 2% to 69% smaller) compared with flat shoe (*p < 0.05*). Double support time decreased significantly for MBT ~12% and rollover shoe ~7% compared to the flat shoe. For both MBT and rollover shoes, there were positive correlations (*r = 0.42-0.60*) between the sole radii and radius of foot-shoe rollover shape (*p < 0.05*).

**Conclusion:**

Wearing MBT and the rollover shoe resulted in more curved foot-shoe, ankle-foot and knee-ankle-foot rollover shapes and faster weight transfer. However, the results also indicate that static sole curve is not the only factor influencing the gait rocker function.

## Background

It is suggested that the goal of walking is to conform to simple curved geometries allowing the body to ‘roll’ forward and thus aid forward progression [1]. This concept takes advantage of the passive dynamics of a rocker-based inverted pendulum [2,3] and has been described in clinical and experimental contexts. Perry [4] for example described heel, ankle, and forefoot rockers operating at initial contact, mid stance and propulsion respectively. A forth ‘toe’ rocker has been suggested, which operates as the limb enters swing [5]. Rocker geometries applied to physical and computational models of walking illustrate the efficiencies of passive dynamic properties [3,6,7].

The so called “rollover shape” has been used to characterise the rollover function of the lower limb during gait. This combines all foot, ankle, knee and shoe movements and deformations into one curved surface over which the body rolls [1,2]. This surface can be characterised by radii of curves fitted to all or discrete sections of the rollover shape [1,8]. Rollover shape is calculated by transforming the centre of pressure into segment coordinate systems, typically the foot or shank [1]. It has been used to design and evaluate lower limb prostheses and orthoses, walking casts/boots and rockers shoes [8]. Footwear with a curved “rollover” sole are prescribed for those who would benefit from reduced motion in the foot [9]. The assumption is that the sole curvature facilitates rolling of the body over the ground reducing the need for movement in the foot. Foot motion might be absent due to disease (e.g. ankle arthritis) [10], or need to be reduced to limit risk of injury (e.g. toe flexion in diabetes) [11]. There is literature describing the effects of rollover footwear on lower limb kinematics, kinetics and to a lesser extent EMG profiles [12-16]. However, apart from anecdotal reports [5], there is a paucity of evidence for how rollover footwear affects the rollover function of the lower limb in walking. We thus have little insight into how this footwear might be of benefit to those with impaired lower limb function and impaired rollover function (e.g. amputees, foot, ankle and knee arthritis).

The only study investigating rollover shape in different rollover footwear [17] demonstrated significant interaction between the two for foot–shoe rollover shapes, but not ankle–foot rollover shapes. They suggested that changes in ankle kinematics occur when wearing different shoes and thus the same rollover shape radius is achieved. However, they did not investigate knee and hip contributions to rollover shape nor effects at these joints due to the footwear. Also in addition to use of a small number of subjects [11], the sole shapes tested were not typical of those commercially available.

Prior work suggests that different radii of sole curvature would have different effects on gait and rollover function [18,19]. Indeed, manufacturers propose that the sole shape is the key distinguishing feature of their products. If true and the static curve of the sole was the only factor influencing the rollover shape, the shoe sole radii and calculated rollover radii should correlate strongly. However, shoe soles deform during gait and this will influence the strength of any relationship. For example, two shoes with identical sole curvature may have very different effects on gait depending upon the sole bending stiffness. Furthermore, soles of rollover footwear do not have a constant radius nor constant material properties across the heel, mid and forefoot. Thus, relationships between static sole radii and rollover shape radii could be complex and be different for different parts of the shoe. To date, the relationship between the sole radii and the rollover function of walking has not been investigated.

Current descriptions of rollover shape do not include the period after heel lift and thus rollover function during propulsion has not been fully characterised. It is in this period that transfer of weight to the contralateral limb is completed. Since the shoe sole is in contact with the ground a curved sole profile might result in a more rapid transfer of weight and thus shorter duration of double limb support.

The aim of this study was three fold. Firstly, to investigate the foot-shoe, ankle-foot and entire lower limb rollover shapes when walking with commercially available rollover footwear compared to flat soled shoes. It was hypothesized that the rollover shapes while walking in rollover footwear would be more curved than those for walking with flat shoes. Secondly, to investigate the effects of rollover footwear on the rollover function between heel-off and toe off. It was hypothesized that the duration of terminal double limb support would significantly decrease when wearing rollover footwear compared to flat shoes due to improved transfer of load forward. Thirdly, to investigate the relationship between the radii of heel, midfoot and forefoot parts rollover footwear soles and the radii of the calculated rollover shape and duration of terminal double limb support. It was hypothesized that the static shape of shoe sole would correlate with roll over function during gait.

## Methods

Subsequent to ethical approval by institutional review board of the University of Salford, 20 healthy subjects (8 female) were recruited (mean age 32.2 years (SD 8.7 years), mean height 1.7 m (SD 0.04 m), mean body mass 70.4 kg (SD13.7 kg) with a mean BMI 24.3 Kg/m2 (SD 4 Kg/m2).

**Figure 1:**
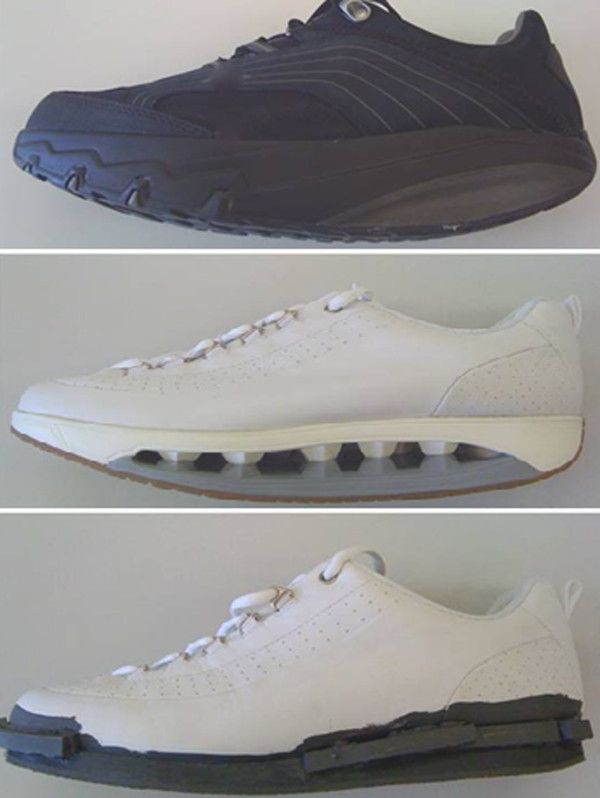
MBT (top) (526 g), rollover shoe rollover footwear (453 g), and weighted flat footwear (453 g) (bottom) (526 g).

Three different shoes of three sole curved profiles were investigated. (i) a MBT shoe (Masai Barefoot Technology), characterized by a stiff rounded sole design in the anterior–posterior direction, with different material under the heel (compliant), and mid and forefoot (stiff); (ii) alternative rollover shoe (a prototype of the Scholl STARLIT shoe), characterized by a solid rounded sole design in the anterior–posterior direction, with consistent materials along the shoe length. The sole is divided into upper and lower layers connected only via multiple hemispheres made from the same material as the sole; (iii) a flat control shoe that had the same weight, upper and last as the alternative rollover shoe and thus differed only in terms of the sole (Figure 1).

### Data collection

Data on the lower limb kinematics and ground reaction force (GRF) were collected during walking on a straight 10 metre course. Participants walked at a self selected speed in each of the three pairs of shoes (randomized order). Kinematic data was collected using 12 cameras (100Hz) (Qualisys motion capture systems, Sweden). Clusters of four reflective markers were positioned on the leg, thigh and posterior pelvis using rigid plates. The position of shoe markers was standardised such that the relationship between the foot and external marker was consistent across the shoe conditions. Shoe markers were located over the first, second and fifth metatarso-phalangeal joints, and the most posterior aspect of the calcaneus. These were located using manual palpation by a single experienced operator to ensure the relationship between the foot and external marker was consistent across the shoe conditions. A relaxed standing trial in the control shoe was used to identify the malleolus, femur epicondyles, greater trochanter, and anterior and posterior superior iliac spines with anatomical markers to establish a suitably anatomical model of the lower limb (Calibrated Anatomical System Technique) [20] and defined the reference position (0°) for all joints. GRF and centre of pressure (COP) data were collected with AMTI force plates (1000 Hz) (AMTI, Watertown, MA, USA) synchronised to motion capture.

### Data processing and analysis

Kinematics and kinetic data exported from Qualysis system were processed using Visual3d software (C-motion, USA). A 4th order Butterworth low-pass filter was applied to marker trajectories and centre of pressure data (cut off 6 and 15 Hz, respectively).

The foot-shoe, ankle–foot and knee–ankle–foot rollover shapes (ROS) were determined by transforming the centre of pressure of the ground reaction force under the shoe from a laboratory-based to foot-shoe, ankle-foot and knee–ankle–foot-based coordinate systems (measured between initial contact and opposite initial contact). The ROS is thus a time progression of the COP of the GRF in the corresponding foot, ankle-foot, and knee-ankle-foot segment coordinate systems. Right and left ROSs were calculated for five stance phases.

The foot-based coordinate system was defined using the medial malleolus (MMAL), lateral malleolus (LMAL) and the head of second metatarsal (D2MT) markers. The origin was located at the ankle joint centre (AJC), the mid-point between MMAL and LMAL. The foot longitudinal axis, y-axis, was the line joining the origin and D2MT. The z-axis was orthogonal to y-axis and pointing upward and the x-axis mutually perpendicular.

The origin of the ankle-foot coordinate system was located midway between medial and lateral femoral epicondyles labelled as knee joint centre (KJC). The vertical axis, z-axis, was along with the projection of line joining AJC and KJC. The x-axis was orthogonal to z-axis and in the plane defined by MMAL, LMAL and z-axis. The y-axis was perpendicular to x- and z- axes. Knee–ankle–foot coordinate system was representing the thigh and shank [1]. The origin was located at the hip joint centre which was determined from the ASIS as a percentage of ASIS-ASIS distance and the coordinates of the greater trochanter marker. The vertical axis joined ankle and hip joint centres. The x-axis was orthogonal to z-axis and in the plane through MMAL, LMAL and trochanter markers. The y-axis was perpendicular to x and z axes.

### Roll-over shapes characterization

Characterization was based on a method in which circular arcs were fitted to all triplets of nonaligned rollover shape data points and then the coordinates of the circumcenters and radius of all circles were averaged. The equation for the circle was used in the non-linear fitting algorithm:

$${\left(X-x_{0}\right)}^{2}+{\left(Z-z_{0}\right)}^{2}=R^{2}$$

where X and Z were the anterior and proximal components, respectively, of the calculated rollover shapes and x_0_, *z*_0_ and R were the horizontal position, vertical position and radius of the center of the average circle, respectively.

The quality of fit (fitting error) was inspected by calculating the root mean square (RMS) of the distance between rollover shape data points (X;Y) and the corresponding point on the arc line of average circle.

**Figure 2:**
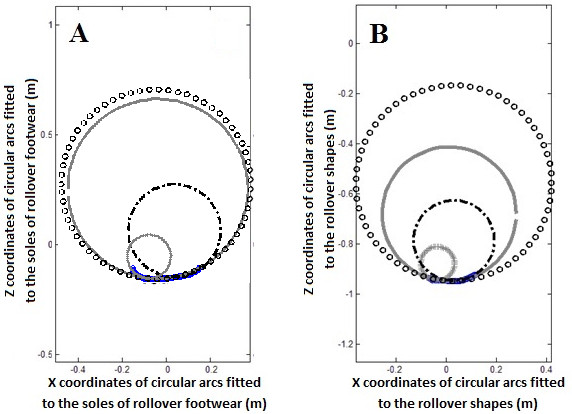
**Average circular arcs of the shoe sole and rollover shapes. A**: Average circular arcs of the whole shoe sole (Solid line) and the first (Plus sign), second (Circle) and final third (Dashed line) of shoe sole. **B**: Average circular arcs of the whole rollover shapes (Solid line) and the first (Plus sign), second (Circle) and final third (Dashed line) of rollover shapes.

Roll over shapes were also divided into three equal parts and the radius of the first, second and final third was calculated using the same method as explained above (Figure 2-B).

All values were first normalized to the subjects’ height (H) in order to compare results with other studies.

Mean roll-over shapes for all three limb systems were found by normalizing X and Z coordinates into 101 samples and then the mean shape coordinates at each point in each shoe condition was calculated. The standard deviation was calculated and plotted at each point as a rectangle of error. Each rectangle of error had a height and width equal to the standard deviations of Z and X coordinates respectively at the normalized sample time. The series of rectangles over the total number of points combined to create a band of error around the mean roll-over shape.

To characterise the rocker function of walking after opposite heel contact, a period that the rollover shape method is unable to characterise, the duration of terminal double limb support was calculated as the time between heel contact on one side and opposite limb toe off. This was normalized to stance time to remove walking speed effects.

For each MBT and rollover shoe size the sagittal plane curved profile of the sole was scribed onto a piece of paper. The shoe was lay on its side and supported so that the sole was at 90° to the table surface. The curves were scanned and a MATLAB script used to extract x and z coordinate of 100 locations along the drawn line. The radius of the curved soles was characterized using the same method as described above for rollover shape. The soles of rollover footwear do not have a constant radius across the heel, mid and forefoot parts of the shoe. Therefore, sole shapes were divided into three equal parts and the radius of the heel, mid and forefoot parts of the sole calculated, as well as the whole sole (Figure 2-A).

SPSS v16 was used to conduct statistical analyses. Normal data distribution was checked by the Shapiro-Wilk test. To compare rollover shapes in the different footwear conditions a Friedman test was used for comparison of multiple groups with repeated measures, to investigate the effects of footwear sole on foot-shoe, foot-ankle and knee–ankle–foot rollover shapes. When the Friedman test was statistically significant, the Bonferroni-adjusted Wilcoxon rank sum test was used for individual paired comparisons. Repeated-measure ANOVA were used to evaluate differences (*p < 0.05*) between footwear conditions in terms of double support time and Bonferroni adjustment for multiple comparisons as a post hoc test employed where differences were found. The relationship between static sole radius and calculated rollover shape radius, and double support time was investigated using Spearman statistics. Multiple regression analysis was conducted to ascertain whether or not the rollover shapes’ radius was related to static sole radius. P-values less than *0.05* were considered statistically significant.

## Results

(Table 1) shows the radius of different parts of curved soles for both MBT and rollover footwear. Results show smallest radius for the heel (most curved area) and largest for the mid foot (flattest area) in both rollover shoes, with MBT having smaller radius overall (more curved). The fitting errors of the whole sole were 0.038 m ± 0.004 m and 0.037 m ± 0.010 m for MBT and rollover shoes, respectively.

**Table 1:** The mean and standard deviations of radius of curved soles (m)

	**Total**	**Forefoot**	**Midfoot**	**Heel**
**MBT**	0.33 ± 0.04	0.18 ± 0.04	0.43 ± 0.40	0.11 ± 0.01
**Rollover shoe**	0.35 ± 0.02	0.32 ± 0.12	0.48 ± 0.16	0.13 ± 0.04

**Figure 3:**
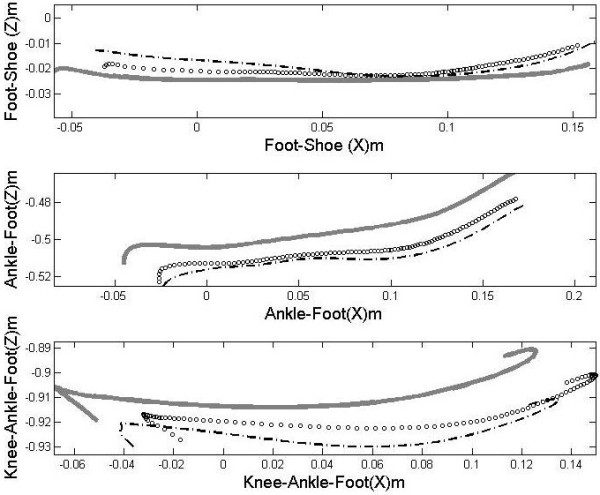
**The foot-shoe, ankle–foot and knee–ankle–foot rollover shapes.** Black dashed = MBT, Black circles = rollover shoe rollover footwear, Grey solid = flat shoe.

**Figure 4:**
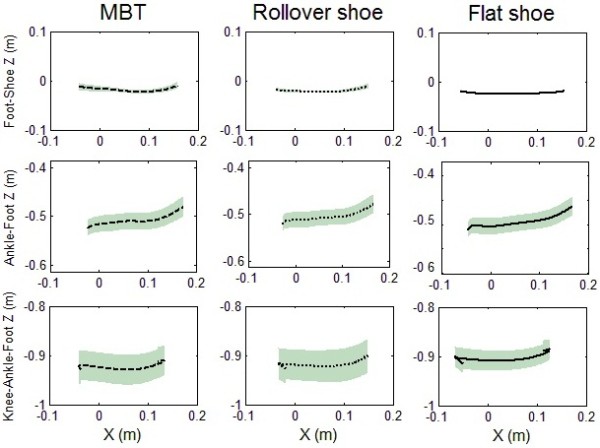
**The mean ± 1standard deviation of foot-shoe, ankle–foot and knee–ankle–foot rollover shapes for MBT, Rollover shoe and Flat shoe.** X and Z values: the anterior and proximal components, respectively, of the calculated rollover shapes.

The mean and standard deviations of radius of curved soles (m) for the whole sole and in each of the heel, midfoot and forefoot areas for MBT and the rollover shoe rollover footwear.

(Figures 3 and 4) shows mean and standard deviation of roll-over shapes for all three limb systems, respectively. (Table 2) shows the radius of average circular arcs for the whole and the middle part (second third) of the rollover shapes, and associated fitting errors.

**Table 2:** The mean radius ± 1SD of the whole and middle part of rollover shapes

**Rollover shapes**		**Condition**	**Mean**	**Mean**	**P Values**
			**Radius ± 1SD (m)**	**Fitting error ± 1SD (m)**	
**Foot- shoe**	**Whole**	MBT	0.23 **±** 0.05	0.034 **±** 0.005	RS**,F**
		Rollover shoe	0.34 **±** 0.07	0.034 **±** 0.005	M**,F**
		Flat shoe	0.76 **±** 0.17	0.036 **±** 0.007	
	**Middle part**	MBT	0.15 **±** 0.04	0.005 **±** 0.002	RS**,F**
		Rollover shoe	0.26 **±** 0.11	0.007 **±** 0.003	M**,F**
		Flat shoe	0.93 **±** 0.48	0.011 **±** 0.005	
**Ankle-foot**	**Whole**	MBT	0.08 **±** 0.04	0.045 **±** 0.005	RS**,F**
		Rollover shoe	0.13 **±** 0.07	0.041 **±** 0.009	M**,F**
		Flat shoe	0.22 **±** 0.06	0.039 **±** 0.009	
	**Middle part**	MBT	0.11 **±** 0.06	0.010 **±** 0.003	RS*,F**
		Rollover shoe	0.16 **±** 0.08	0.013 **±** 0.004	M*,F**
		Flat shoe	0.37 **±** 0.24	0.016 **±** 0.005	
**Knee–ankle–foot**	**Whole**	MBT	0.14 **±** 0.04	0.037 **±** 0.009	RS**,F**
		Rollover shoe	0.16 **±** 0.05	0.036 **±** 0.009	M*
		Flat shoe	0.17 **±** 0.04	0.038 **±** 0.011	
	**Middle part**	MBT	0.17 **±** 0.06	0.015 **±** 0.005	RS**,F**
		Rollover shoe	0.24 **±** 0.12	0.017 **±** 0.004	M**,F*
		Flat shoe	0.27 **±** 0.11	0.017 **±** 0.006	

The mean radius ± 1Standard Deviation (SD) of the whole and middle part of rollover shapes (Normalized to body height) and the mean fitting error ± 1SD. *RS* Rollover Shoe, *M* MBT, *F* Flat shoe; ** = *p <0.001*, * = *p < 0.05*.

The radius of average circular arcs of the whole and middle part of foot–shoe rollover shapes, ankle-foot rollover shapes and knee–ankle–foot rollover shapes were significantly different between shoe conditions (*p < 0.05*). The only exception was in the case of whole knee-ankle-foot rollover shapes where there was no significant differences between the alternative rollover shoe and flat shoe .Pairwise comparisons indicated the mean radius of the average circular arcs of the whole and middle part of all rollover shapes were significantly smaller for MBT (ranging from 12% to 81%) and the rollover shoe (ranging from 2% to 69%) compared to the flat shoe, and for MBT compared to rollover shoe (ranging from 11% to 37%) (Table 2, Figure 3).

For the first third, there was significant difference between shoe conditions only in the case of ankle-foot rollover shapes (*p < 0.05*), where the radius of average circular arcs was significantly larger for MBT (0.09 m **±** 0.07 m) compared to the rollover shoe (0.05 m **±** 0.03 m) and the flat shoe (0.05 m **±** 0.03 m). The fitting errors were 0.020 m ± 0.004 m, 0.021 m ± 0.004 m and 0.021 m ± 0.004 m for MBT, the rollover shoe and the flat shoe, respectively.

For the final third, the radius of average circular arcs of the foot–shoe rollover shapes and ankle-foot rollover shapes were significantly different for the rollover shoe (0.19 m **±** 0.09 m, 0.14 m **±** 0.10 m, respectively) compared to MBT (0.09 m **±** 0.04 m; 0.12 m **±** 0.10 m, respectively) and the flat shoe (0.15 m **±** 0.10 m; 0.11 m **±** 0.10 m, respectively), and for MBT compared to flat shoe only in the case of the foot–shoe rollover shapes (*p < 0.05*). The fitting errors were between 0.006 m ± 0.002 m and 0.010 m ± 0.003 m.

Double support time decreased significantly (*p < 0.001*) for MBT (15.3% ± 1.2%) and rollover shoe (16.2% ± 1.2%) compared to the flat shoe (17.4% ± 1.3%). The double support time of MBT was lower than that of rollover shoe condition (*p < 0.001*).

For the rollover shoe, there was only a significant positive correlation between the whole static sole radius and the radius of foot-shoe rollover shape in the final third (forefoot part) (*r = 0.60; p < 0.001*). Thus there was some evidence that the more curved the whole sole, the more curved the final third of the radius of foot-shoe rollover shape. The result of regression analysis showed that the whole static rollover shoe radius could explain 48% of variance in foot-shoe rollover shapes radius in the final third (*f = 11.37, p = 0.002*). There was also no statistically significant relationship between the static sole radius and the normalized double support time.

For the MBT shoes, there was a significant positive correlation between the radius of foot-shoe rollover shape and the whole static sole radius (*r = 0.42; p = 0.012*). Thus there was some evidence that the more curved the whole sole, the more curved the radius of foot-shoe rollover shape. No relationship was found between the static sole radius and the radius of ankle–foot and knee–ankle–foot rollover shapes (*r < 0.29, p > 0.05*). The result of regression analysis showed that the whole static MBT radius could explain 45% of variance in foot-shoe rollover shapes radius (*f = 8.39, p = 0.007*). There was no statistically significant relationship between the static sole radius and the normalised double support time.

## Discussion

The purpose of this study was to better understand how the rollover function of walking is affected by rollover footwear. As hypothesized the radius of foot-shoe, ankle-foot and knee–ankle–foot rollover shapes were significantly reduced when wearing MBT and the alternative rollover shoe. These effects could be useful for people who have lost normal strategies for sagittal plane motion in lower limb joints because this footwear might replace some of the lost capacity for “rolling over” the ground in a normal manner. For example, in cases of surgical ankle arthodesis, the loss of ankle motion could lead to very large radii of rollover shape and loss of normal rollover function. Increased compensatory motion at adjacent joints will be required if the pre-existing rollover shape is to be maintained. Indeed, midfoot arthritis is a major complication of ankle arthrodesis suggesting this compensation does occur [21]. Therefore, use of rollover footwear might negate the need for this midfoot compensation and reduce the risk of midfoot arthritis post ankle arthrodesis.

Rollover shape radius of the whole and middle part was reduced more in the MBT than the alternative rollover shoe. This could be due to more curved profile of MBT shoes soles (Table 1). The heel area of the MBT soles was also more compressible than the alternative rollover shoe and greater deformation in this area might lead to a smaller ROS radius.

The radius of the ankle-foot ROS changed appreciably in response to rollover footwear conditions. This is in contrast with previous studies which have reported that ankle–foot rollover shapes are robust to perturbations in walking speed [1], footwear heel height [22,23] and rocker radius [17]. Significant changes of the ankle-foot rollover shapes in our study can be explained by more severe and complex nature of the shoe interventions. We suggest that the ankle and foot joints could not compensate for the perturbations in ankle-foot function imposed by the rollover shoes and therefore the ankle-foot ROS changed [17].

The knee-ankle-foot rollover shape represents the rollover function of the entire lower limb system. The radius for the middle third, where there were no inflection points in data and fitting errors were significantly less than the whole shape, were 0.17, 0.24 and 0.27 times participants’ height for MBT, the alternative rollover shoe and flat shoes, respectively. Based on an assumption that the entire lower limb is 0.53 times participant height [24] this radius is the equivalent to 0.32, 0.45 and 0.51 times the leg length in the case MBT, rollover shoe and flat shoes, respectively. It has been reported that healthy individuals walk with a minimum metabolic rate when the rocker radius is approximately 0.3 times leg length [18] and 0.3 has also been advocated for human like walking machines [3]. The values for rollover footwear, especially MBT, are closer to the proposed optimal radius of 0.3 compare to the flat shoe and thus perhaps lead to reduced metabolic cost of walking. However, our own work using the same footwear and subjects reported here found no significant change in energy cost of walking in rollover footwear [12].

In the middle third, points’ distribution is known to cover almost a perfect circular arc with no outliers or deflection point and therefore it could be a better representative of rollover shapes. The results of fitting quality also showed showed better fit for the middle third compared to the whole rollover shapes.

The radius of the rollover shoes did not have a very strong relationship with the rollover shapes characterising lower limb function. This is because static shoe sole geometry does not reflect the shape to which the shoe sole deforms under load. This outcome questions the assertion of manufacturers that precise design and static geometric features of a rollover shoe sole relate precisely to effects on gait and posture. Instead, the effect of their footwear is a combination of the static sole geometry, the dynamic material properties of the sole and the user and task specific loads that are applied.

The ROS approach is unable to characterise rollover function after heel off [1]. We investigated rollover function in this period using the duration of double limb support time as a measure of load transfer. Rollover footwear reduced the time of double limb support suggesting a faster weight transfer to the opposite limb compared to walking with flat shoes.

One of the limitations of the present study is that the radius of rollover shapes was the only aspect calculated and investigated, although the other aspects of these shapes such as arc length, position, and orientation might also have an important effect. Thus we are not here able to offer guidelines for optimised sole designs related to specific rehabilitation technologies. Another limitation of our study is that the tested shoes were different from each other in terms of sole material properties such as bending stiffness and material density which could have influence on our findings.

## Conclusion

Wearing MBT and the rollover shoe resulted in more curved foot-shoe, ankle-foot and knee-ankle-foot rollover shapes and faster weight transfer which could be useful for people who have lost normal rollover function and strategies for sagittal plane motion in lower limb joints. This includes individuals with ankle arthritis, spasticity in posterior calf muscles, or ankle arthrodesis. The results also indicate that static sole curve is not the only factor influencing the gait rocker function.

## Consent

Written informed consent was obtained from the patient for the publication of this report and any accompanying images.

## Competing interests

The kinematic data used in the paper was originally collected as part of experimental tests to evaluate the effect of various footwear products on gait (under review elsewhere). That work was funded by SSL International, who own the Scholl brand of footcare/footwear. Their staff played no part in the work other than agreeing its general objectives. None of the authors received financial benefit from the work in the original study, nor this paper. No author has received any personal financial benefit nor has any personal commercial association with the company, nor the owners of the other footwear tested in this study. The author(s) declare that they have no other competing interests.

## Authors’ contributions

SF and BR were the primary researchers involved in all aspects of the research and writing. CN provided senior research advice and support for all aspects of work. This included detailed contributions to the research design, the nature and management of data and its analysis, and paper writing. All authors read and approved the final manuscript.
